# Ultrasonographic assessment of carpal tunnel syndrome of mild and moderate severity in diabetic patients by using an 8-point measurement of median nerve cross-sectional areas

**DOI:** 10.1186/1471-2342-12-15

**Published:** 2012-07-07

**Authors:** Shu-Fang Chen, Chi-Ren Huang, Nai-Wen Tsai, Chiung-Chih Chang, Cheng-Hsien Lu, Yao-Chung Chuang, Wen-Neng Chang

**Affiliations:** 1 Department of Neurology, Kaohsiung Chang Gung Memorial Hospital and Chang Gung University College of Medicine, Tai-Pei road, Kaohsiung 833, Taiwan; 2 Department of Biological Science, National Sun Yat-Sen University, Kaohsiung, Taiwan

## Abstract

**Background:**

Using high-resolution ultrasonography (US) to measure the median nerve cross-sectional areas (CSAs) such as in the “inching test” conducted in nerve conduction studies is a valuable tool to assess carpal tunnel syndrome (CTS). However, using this US measurement method to assess the median nerve CSA in diabetic patients with CTS has rarely been reported. Therefore, we used this US measurement method in this study to measure median nerve CSAs and to compare the CSAs of idiopathic, diabetic and diabetic polyneuropathy (DPN) patients with CTS.

**Methods:**

124 hands belonging to 89 participants were included and assigned into four groups: control (32), idiopathic (38), diabetic (38) and DPN (16) CTS. In the latter two groups, only patients with mild and moderately severe CTS were included. The median nerve CSAs were measured at 8 points marked as *i*4, *i*3, *i*2, *i*1, *w*, *o*1, *o*2, and *o*3 in the inching test. The measured CSAs in each group of participants were compared.

**Results:**

Compared with the CSAs of the control group, enlarged CSAs were found in the idiopathic, diabetic and DPN CTS groups. The CSAs were larger at *i*4, *i*3 and *i*2 in the diabetic CTS group compared to the idiopathic CTS group. The CSAs measured at the *i*1 and *w* levels of the DPN CTS group were smaller than those of the diabetic CTS group. In the diabetic CTS group, the cut-off values of CSAs measured at the inlet, wrist crease, and outlet were 15.3 mm^2^, 13.4 mm^2^ and 10.0 mm^2^, respectively, and 14.0 mm^2^, 12.5 mm^2^ and 10.5 mm^2^, respectively, in the DPN CTS group.

**Conclusions:**

Compared with the median nerve CSAs of the control and idiopathic CTS groups, the median nerve CSAs of the diabetic patients with CTS were significantly enlarged. However, compared with the diabetic CTS group, the CSAs were significantly smaller in the DPN CTS group. This US 8-point measurement method can be of value as an important complementary tool for CTS studies and diagnosis among diabetic patients.

## Background

Carpal tunnel syndrome (CTS) is a common entrapment neuropathy with prevalence rates of 2% in the general population, 14% in diabetic patients without diabetic polyneuropathy (DPN), and 30% in diabetic patients with DPN [1,2]. The diagnosis of CTS is usually based on clinical symptoms as well as the results of nerve conduction studies (NCS). However, because of diabetic hand syndromes, the diagnosis of CTS in patients with diabetes can be difficult if using clinical symptoms and NCS with various comparative tests [3-7] alone. Ultrasonography (US) is a non-invasive and easily performed procedure for median nerve morphology measurement. Based on the findings of median nerve cross-sectional area (CSA) enlargement in the carpal tunnel, US can be used to confirm CTS with a high degree of accuracy [8-10]. US has been used in clinical studies on diabetic neuropathy [11,12], however its use in the clinical evaluation of CTS in diabetic patients has not been reported in the literature. In our previous study, we reported the results of an US study [10] for the evaluation of idiopathic CTS using the same 8-point measurements of the median nerve CSAs from inlet to outlet as in the “inching test” of antidromic sensory studies using 1-cm increments of the median nerve [13]. Although there were some limitations to the study, the results showed the value of this US measurement method as an important complementary tool to confirm CTS. Therefore we used this US measurement method in this study to measure median nerve CSAs, and to compare the measured CSAs of idiopathic, diabetic and DPN patients with CTS.

## Methods

### Patients

This prospective case–control study was carried out over a period of four years (2006–2009), and was approved by the Ethics Committee of Chang Gung Memorial Hospital (IRB 100-1390B). In this study, the following procedures were used to enroll the study cases: first, those who had signs and symptoms fulfilling the clinical diagnostic criteria of CTS were referred by the authors to undergo NCS and US studies; then, those who fulfilled the inclusion criteria of idiopathic CTS, diabetic CTS or DPN CTS were further considered for enrollment, and finally, those who agreed to sign informed consent forms were enrolled into this study. Normal controls were also recruited.

In this study, diabetic patients were defined as those who had symptoms (polyuria, polydipsia, and unexplained weight loss) of diabetes mellitus (DM) plus a casual plasma glucose concentration ≥ 200 mg/dL, or a fasting plasma glucose level of ≥ 126 mg/dL on at least two occasions, or a plasma glucose level of ≥ 200 mg/dL at two hours for a 75-g oral glucose tolerance test (OGTT) [14]. In the control and idiopathic CTS groups, DM was excluded by a fasting serum glucose level < 100 mg/dl, or a two-hour postprandial serum glucose level < 110 mg/dl and no clinical symptoms of DM. Serum glycohemoglobin (HbA1C) levels of all enrolled participants were measured. Patients with other systemic diseases including gout, rheumatic arthritis, thyroid disease, renal disease, hepatic disease, abnormal serum cortisol levels or elevated serum antinuclear antibodies were excluded by blood tests and clinical history. None of the participants had a history of wrist surgery or fracture, and none had a history or any clinical evidence of neurologic disorders (e.g. ulnar neuropathy, radiculopathy, polyneuropathy (not DM related), myelopathy, or stroke) that may have resulted in numbness or paresthesia of the hand. Participants with a variant of carpal tunnel such as accessory muscles, bifid median nerve and persistent median artery found by US were also excluded. In addition, none of the female participants were pregnant at the time of the study.

### Definition of clinical CTS

Clinical CTS was defined according to the criteria of The American Academy of Neurology practice parameters [15,16] as follows:

1. Paresthesia, pain, swelling, weakness, or clumsiness of the hand provoked or worsened by sleep, sustained hand or arm position, or repetitive action of the hand or wrist that is mitigated by changing posture or by hand shaking.

2. Sensory deficits in the median nerve innervated region of the hand.

3. Motor deficit or hypotrophy of the median nerve innervated thenar muscles.

4. Positive provocative clinical tests (positive Phalen’s maneuver and/or Tinel’s sign).

Clinical CTS was defined as the fulfillment of criterion 1 and one or more of the other criteria.

### Clinical definition of polyneuropathy

The symptoms and signs of suspected DPN were examined according to the recommendations of the American Academy of Electrodiagnostic Medicine (AAEM) [17]. A combination of neuropathic symptoms, neuropathic signs and abnormal electrodiagnostic studies provides the diagnosis of distal symmetric polyneuropathy. In this study, an electrodiagnostic abnormality plus at least one sign and one symptom were sine qua non for the confirmation of the presence of polyneuropathy. The neuropathic symptoms included sensory symptoms (distal numbness, burning, prickling paresthesia, dysesthesia and allodynia) and/or motor symptoms (decreased sensibility on the distal lower extremity, distal muscle weakness or atrophy). The neuropathic signs included an absent or decreased ankle deep tendon reflex, distal sensory decrease or absence, and distal weakness and muscle atrophy. Abnormal electrodiagnostic studies included at least a sural, or peroneal and one median or ulnar nerve dysfunction, however entrapment lesions were excluded

### Electrophysiologic methods

**Figure 1:**
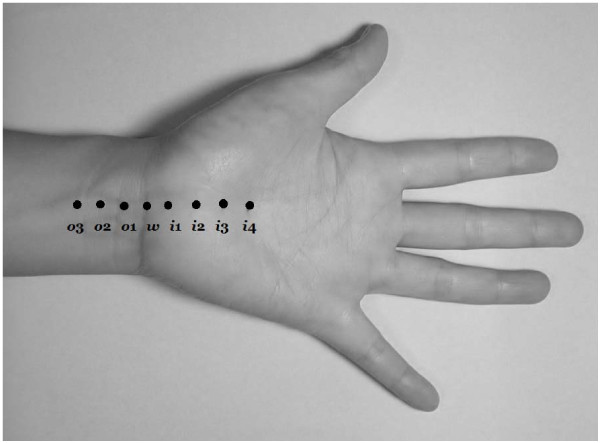
**The 8 points (*i*4, *i*3, *i*2, *i*1, *w*, *o*1, *o*2, and *o*3) for recording in both the “inching test” and ultrasonography.***i*4, *i*3, *i*2, *i*1 represent levels at 4, 3, 2, and 1 cm distal to the wrist crease in the inlet of the carpal tunnel; *w* represents the level of the wrist crease and *o*1, *o*2, and *o*3 represent levels at 1, 2, and 3 cm proximal to the wrist crease in the outlet of the carpal tunnel.

NCS was performed for all participants according to the recommended protocol of the AAEM [18] using a Nicolet Viking Select system (Nicolet Biomedical Inc. Madison, USA). The comparative tests and the cut-off points were as follows [19-23]: 1) median-ulnar sensory conduction between the wrist and ring finger; 2) median sensory nerve conduction comparison between the wrist and palm; 3) median-radial sensory conduction between the wrist and thumb; and 4) antidromic sensory test using 1-cm increments of the median nerve; 5) median nerve distal sensory latency < 3.4 ms; 6) median nerve distal motor latency over the thenar < 4.2 ms; 7) a difference between the median and ulnar nerve distal sensory latencies < 0.4 ms; 8) transcarpal median motor conduction velocity < 40.6 ms; and 9) antidromic sensory testing using 1-cm increments of the median nerve < 0.4 ms. The locations of the inching test were as shown in Figure 1, with the wrist crease as the zero reference point extending proximally by 3 cm and distally by 4 cm. In total, eight points (*i*4, *i*3, *i*2, i1, *w*, *o*1, *0*2, *0*3) were marked in the subsequent inching test. According to the results of the NCS, the CTS hands were categorized into six groups of severity [23]: negative, minimal, mild, moderate, severe and extreme. In this study, only those belonging to the mild (abnormal digit/wrist sensory nerve conduction velocity and normal distal motor latency) and moderate (abnormal digit/wrist sensory nerve conduction velocity and abnormal distal motor latency) NCS groups were included in the final analysis.

NCS for polyneuropathy detection was also performed according to the study protocol of the AAEM [17]. Sural, tibial, ulnar and peroneal nerves were also examined to determine whether or not the included participants fit the diagnostic criteria of polyneuropathy. The segmental tests (ulnar nerve at elbow and peroneal nerve at the fibula head) were also performed to exclude entrapment neuropathy.

The normal limits of NCS of the abovementioned nerves other than the median nerve were as follows: ulnar nerve (distal motor latency < 3.4 ms, motor conduction velocity > 52 m/s, sensory conduction velocity > 44 m/s, compound muscle action potential > 5.5 mV, sensory nerve action potential > 9 μV), peroneal (distal latency < 5.5 ms, conduction velocity > 42 m/s, compound motor action potential > 2.1 mV), tibial nerve (distal latency < 6.4 ms, conduction velocity > 41 m/s, compound motor action potential > 4.7 mV), and sural nerve (conduction velocity > 38 m/s, sensory nerve action potential > 5 μV).

### Ultrasound assessment technique

The US assessment technique we used in this study has been previously described [10]. In brief, high-resolution US were performed using a scanner with a 12/5-MHz linear array transducer for the carpal tunnel study (Philips HDI 5000; Philips Medical Systems, Bothell, WA, USA). US examinations were performed on the same day as the NCS. During the examination, the patient sat in a comfortable position facing the examiner, with the measured forearm resting on the table, the palm supine, and fingers semi-extended in the neutral position [24]. The median nerve was imaged in a longitudinal scan first, placing the US probe at the midline between the radius and ulna with the center of the probe at the distal wrist crease, to obtain an initial general overview of the median nerve which was then used to assist the examiner in order to obtain optimal axial (cross-sectional) images. Then a transverse scan, keeping the probe directly perpendicular to the long axis of the median nerve in order to ensure that the area measured indeed reflected a CSA, was performed to record the CSA (calculated by continual tracing of the nerve circumference, excluding the hyperechoic epineurial rim) and elliptical (the transverse and the anteroposterior) diameters. The measurements were performed from the inlet of carpal tunnel to the distal segment of the forearm, as shown in Figure 1, at the 8 points (*i*4, *i*3, *i*2, i1, *w*, *o*1, *0*2, *0*3) [10].

### Study patient groups

In total, 124 hands belonging to 89 participants were enrolled in this study, and they were divided into four groups as follows:

1. Control group (22 participants, 32 hands):

Healthy volunteers who had no clinical or electrophysiologic evidence of CTS or other neurologic disorders.

2. Idiopathic CTS group (30 participants, 38 hands):

Those who had clinical and electrophysiologic evidence of CTS, but no other medical or neurologic disorders that may have resulted in numbness or paresthesia of the hands.

3. Diabetic CTS group (26 participants, 38 hands):

Those who had clinical symptoms and findings of CTS, and also fulfilled the electrophysiologic criteria of CTS. They did not have clinical or electrophysiologic evidence of polyneuropathy, ulnar or radial neuropathy.

4. DPN CTS group (11 participants, 16 hands):

Diabetic patients who had clinical symptoms and findings of CTS and also fulfilled the electrophysiologic criteria of CTS. They also had clinical and electrophysiologic evidence of polyneuropathy.

Among the enrolled CTS patients, bilateral hand involvement was noted in 11 patients of the idiopathic CTS group, 7 in the diabetic CTS group and 3 in the DPN CTS group.

### Statistical analysis

The data were all presented as mean ± standard deviation (median, range) for statistical analysis. Comparisons between the demographic data of the control, idiopathic, diabetic CTS and DPN CTS groups were made using the Kruskal-Wallis test for continuous variables including age, body weight (BW), body height (BH), body mass index (BMI). The chi-square test was used for the categorical variables including sex. To evaluate the differences in CSA values measured at the 8 points between the idiopathic, diabetic and DPN CTS hands, the Mann–Whitney U test was used. Significance was set at *p* < 0.05 in the Mann–Whitney U test and Kruskal-Wallis test. The area under the receiver operating characteristic (ROC) curves and the cut-off-values of CSA were calculated for the idiopathic, diabetic and DPN CTS groups. The Statistical Package for Social Sciences software (SPSS Inc., version 13.0 for Windows) was used for all statistical analyses.

## Results

The demographic data of the 89 participants (142 hands) are shown in Table 1. There were no significant differences in age, gender and BW among the control, idiopathic, diabetic and DPN CTS groups. Compared with the control group, the BH was shorter in the diabetic and DPN CTS groups; however the BMI was higher in the idiopathic, diabetic and DPN CTS groups. If the control group was not included for analysis, differences in MBI values among the idiopathic, diabetic and DPN CTS groups were not significant (*p* = 0.165). There were no significant differences in HbA1c values between the diabetic and DPN CTS groups.

**Table 1:** Demographic data of the 89 participants (124 hands)

	**Control hands**	**Idiopathic CTS hands**	**Diabetic CTS hands**	**DPN CTS hands**	***p *value**
Numbers	32	38	38	16	
Sex	9 M/23 F	9 M/29 F	9 M/29 F	4 M/12 F	0.971^**﹢**^
Age (yrs)	56.5 ± 8.0 (54.5, 48.0-76.0)	59.2 ± 9.3 (57.0, 44.0-83.0)	58.6 ± 9.4 (56.0, 38.0-76.0)	61.0 ± 7.8 (63.0, 50.0-73.0)	0.365
BH (cm)	160.6 ± 8.6 (159.0, 151.0-184.0)	157.0 ± 5.1 (157.0, 147.0-167.0)^**※**^	155.3 ± 5.3 (154.0, 146.0-170.0)	151.7 ± 4.8 (150.0, 143.0-160.0)	0.002^*^
BW (kg)	61.8 ± 9.6 (60.0, 51.3-96.0)	63.3 ± 9.5 (62.0, 47.0-82.0)	68.0 ± 12.0 (67.0, 45.0-92.0)	61.6 ± 10.9 (60.0, 50.0-88.0)	0.099
BMI kg/m^2^	23.9 ± 2.3 (24.2, 19.7-28.4)	25.6 ± 3.2 (24.9, 20.3-31.6)	28.0 ± 4.5 (27.2, 19.7-35.9)	26.8 ± 4.8 (25.6, 21.4-39.1)	0.002^*^
HbA1c (%)	5.6 ± 0.4 (5.6, 5.0-6.2)	5.8 ± 0.3 (5.8, 5.3-6.3)	7.3 ± 1.0 (7.2, 5.7-9.5)	7.9 ± 3.0 (6.9, 6.1-18.9)	0.904

CTS = carpal tunnel syndrome; DPN = diabetic polyneuropathy; BH = body height; BW = body weight; BMI = body mass index; M = male; F = female.

The data shown are mean ± standard deviation (SD) (median, range).

^*^ = *p* < 0.05 (significant difference among the normal control, idiopathic, diabetic and DPN CTS hands) by the Kruskal-Wallis test.

^**﹢**^ = a comparison of the sex difference and symptom duration among the control, idiopathic, diabetic and DPN CTS hands by the Chi-Square test.

^**※**^ = a comparison of the BH between the control and the idiopathic CTS hands by the Mann–Whitney U test showed no significant difference.

The CSAs measured at the 8 points (*i*4, *i*3, *i*2, *i*1, *w*, *o*1, *o*2, *o*3) of the four groups of participants are listed in Table 2. The measured CSAs were larger at *i*4, *i*3 and *i*2 of the diabetic group than those of the idiopathic CTS group. Compared with the diabetic group, the CSAs measured at the *i1* and *w* levels of the DPN group were smaller.

**Table 2:** The measured CSAs of the control, idiopathic, diabetic and DPN groups

	**Control hands (n = 32)**	**Idiopathic CTS hands (n = 38)**	**Diabetic CTS hands (n = 38)**	**DPN CTS hands (n = 16)**	***p *value**^**1**^	***p *value**^**2**^	***p *value**^**3**^
***i4***	12.6 ± 3.0 (12.0, 6.0-19.0)	17.5 ± 6.4 (16.5, 8.0-39.0)	20.4 ± 5.4 (19.5, 13.0-34.0)	20.9 ± 13.8(15.5, 8.4-62.0)	0.015*	0.109	0.000^*^
***i3***	12.2 ± 2.9 (12.0, 6.0-19.0)	16.3 ± 5.8 (15.5, 8.0-33.0)	19.3 ± 5.5 (19.0, 10.0-31.0)	19.0 ± 9.1 (15.5, 8.4-43.0)	0.015*	0.310	0.000^*^
***i2***	11.5 ± 2.5 (11.0, 7.0-18.0)	12.5 ± 3.7 (12.0, 6.0-29.0)	14.2 ± 3.9 (13.5, 9.0-24.0)	13.6 ± 5.9 (11.5, 9.0-34.0)	0.026*	0.242	0.010^*^
***i1***	10.8 ± 2.1 (10.2, 7.0-17.0)	14.0 ± 4.8 (13.0, 8.0-28.0)	15.5 ± 4.4 (14.8, 7.7-26.1)	12.7 ± 3.1 (13.0, 7.0-21.0)	0.105	0.043^*^	0.000^*^
***w***	11.8 ± 2.6 (11.0, 8.0-19.0)	16.2 ± 4.5 (15.0,11.0-31.0)	17.9 ± 6.6 (16.0, 8.1-45.8)	14.8 ± 4.7 (13.5, 8.0-28.0)	0.217	0.039^*^	0.000^*^
***o1***	10.6 ± 2.4 (10.0, 6.0-16.0)	14.0 ± 3.7 (13.4, 8.0-23.0)	15.4 ± 5.9 (14.0, 7.0-42.8)	14.3 ± 5.5 (13.0, 7.0-30.0)	0.237	0.458	0.000^*^
***o2***	11.0 ± 2.6 (10.5, 7.0-17.0)	12.2 ± 2.6 (12.0, 8.0-19.5)	13.8 ± 4.0 (13.0, 8.6-29.0)	12.8 ± 5.3 (12.5, 7.4-29.0)	0.090	0.181	0.006^*^
***o3***	10.0 ± 2.4 (9.5, 7.0-15.0)	10.8 ± 2.2 (10.4, 6.0-16.0)	12.4 ± 3.5 (11.7, 7.0-20.0)	12.2 ± 4.0 (10.5, 8.7-25.0)	0.060	0.601	0.017^*^

CTS = carpal tunnel syndrome; CSA = cross-sectional area; DPN = diabetic polyneuropathy.

The data shown are mean ± standard deviation (SD) (median, range).

Unit of CSA = mm^2^.

*i4*, i3, *i2*, *i1*, *w*, *o1*, *o2*, and *o3* = the 8 points marked.

***p *value**^**1**^ **=** a comparison between the idiopathic and the diabetic CTS hands using the Mann–Whitney U test.

***p *value**^**2**^ **=** a comparison between the diabetic and the DPN CTS hands using the Mann–Whitney U test.

***p *value**^**3**^ **=** a comparison among control, idiopathic CTS, the diabetic and the DPN CTS hands using the Kruskal-Wallis test.

* = *p* < 0.05.

**Figure 2:**
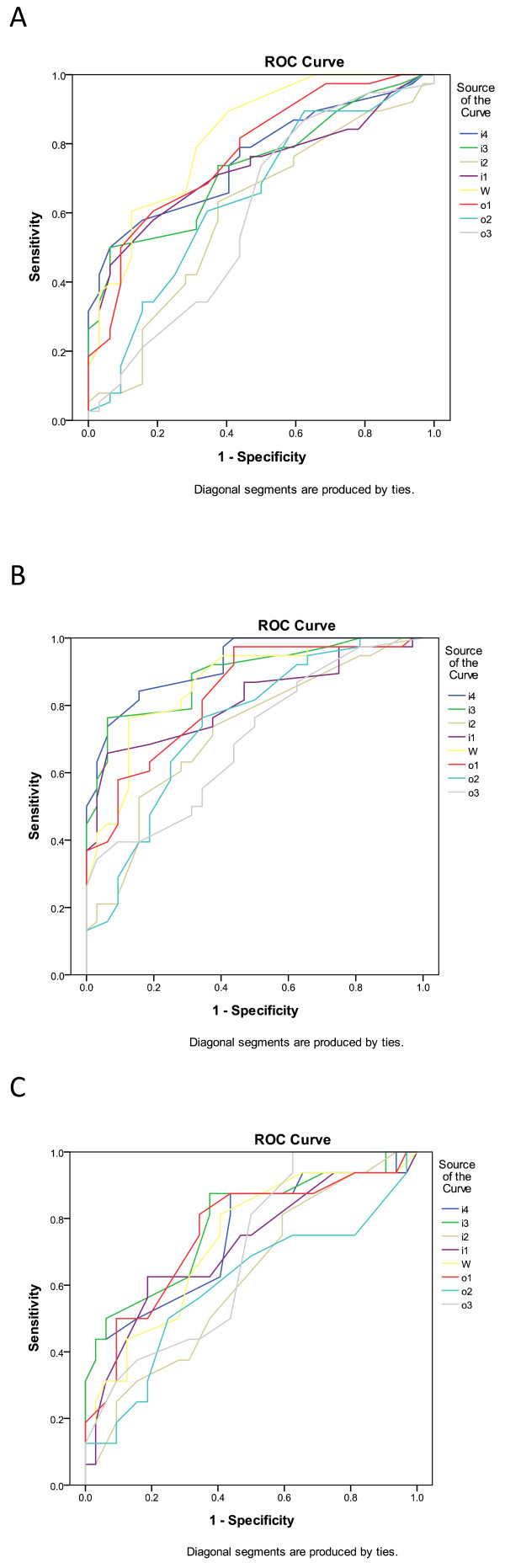
ROC curves of the cut-off values of cross-sectional areas (CSA) of the idiopathic (A), diabetic (B) and diabetic polyneuropathy (DPN) (C) carpal tunnel syndrome (C).

Table 3 and Figure 2 show the cut-off values and corresponding sensitivities and specificities of CSAs in the diagnosis of CTS in the idiopathic, diabetic and DPN groups. These cut-off values were derived from a comparison with the CSAs of the control group. In the idiopathic group, the cut-off values of CSAs measured at the inlet, wrist crease, and outlet were 13.0 mm^2^, 12.5 mm^2^ and 9.5 mm^2^, respectively; in the diabetic group 15.3 mm^2^, 13.4 mm^2^ and 10.0 mm^2^, respectively; and 14.0 mm^2^, 12.5 mm^2^ and 10.5 mm^2^, respectively, in the DPN group. The largest CSA cut-off value at the *w* level was in the diabetic group. At the outlet levels, there were no significant differences in cut-off values between the control and the idiopathic group, however there were statistically significant differences between the control (CSA cut-off value = 9.5 mm^2^) and diabetic group (CSA cut-off value = 10.0 mm^2^), and between the control and the DPN group (CSA cut-off value = 10.5 mm^2^).

**Table 3:** The cut-off values of CSA of the idiopathic, diabetic and DPN CTS groups (compared with the control group)

**Location**	**Idiopathic CTS group**					**Diabetic CTS group**					**DPN CTS group**				
	**Sen.**	**Spec.**	**Area**	**Cuff-off value of CSA**	**Sig.**	**Sen.**	**Spec.**	**Area**	**Cuff-off value of CSA**	**Sig.**	**Sen.**	**Spec.**	**Area**	**Cuff-off value of CSA**	**Sig.**
*i4*	73.7	59.4	0.760	13.0	0.000*	84.2	84.4	0.921	15.3	0.000*	62.5	59.4	0.750	14.0	0.005*
*i3*	73.7	62.5	0.734	12.8	0.001*	76.3	93.7	0.889	15.3	0.000*	62.5	68.7	0.784	14.0	0.001*
*i2*	63.2	62.5	0.602	11.4	0.142	63.2	71.9	0.722	12.4	0.001*	50.0	62.5	0.620	11.5	0.179
*i1*	71.1	62.5	0.727	11.5	0.001*	76.3	62.5	0.813	11.4	0.000*	62.5	62.5	0.716	11.5	0.016*
*w*	78.9	68.7	0.824	12.5	0.000*	81.6	71.9	0.859	13.4	0.000*	62.5	68.7	0.731	12.5	0.010*
*o1*	68.4	65.6	0.777	11.5	0.000*	81.6	65.6	0.832	11.2	0.000*	75.0	65.6	0.757	11.7	0.004*
*o2*	60.5	65.6	0.647	11.5	0.035*	76.3	65.6	0.744	11.0	0.000*	56.3	65.6	0.600	11.5	0.265
*o3*	73.7	50.0	0.598	9.5	0.159	68.4	56.2	0.698	10.0	0.004*	50.0	56.2	0.688	10.5	0.035*

CSAs = cross-sectional areas; DPN = diabetic polyneuropathy; Sen. =sensitivity; Spec. =specificity; Sig. =significance; ROC = Receiver Operating Characteristic; CTS = carpal tunnel syndrome; NCS = nerve conduction study.

*i4*, *i3*, *i2*, *i1*, *w*, *o1*, *o2*, and *o3* = the 8 points marked.

Unit of CSA = mm^2^; Sen. = %; Spec. = %.

* = Sig. < 0.05 (asymptotic significance under the ROC curve).

## Discussion

In clinical practice, it is difficult to distinguish CTS from other neuropathic syndromes in diabetic patients, even when electrodiagnostic tests are also applied for such purposes [2,5,6]. An enlargement of median nerve CSA measured in CTS hands is a well established US finding [8-10], and with multiple-level CSA measurements, the complementary role of US in the diagnosis of CTS becomes more valuable [10]. The same US findings were also noted in the present study which showed enlarged CSAs in patients with idiopathic CTS as well as in those with diabetic CTS and DPN CTS. There are reports [11,12] of US studies of peripheral nerves in diabetic patients, however using US to measure the CSAs at 8 points such as in the “inching test” to assess CTS in diabetic patients has not been reported before.

The study results showed that the measured CSAs in both the diabetic and DPN groups were larger than those measured in the idiopathic and control groups. The BMIs of patients of the CTS groups were significantly larger than those of patients in the control group. It has been proposed that a higher BMI may increase the incidence of symptomatic CTS [3], however a significant correlation with median nerve CSA measured at the wrist level was not found in the study of Werner et al. [25]. It may deserve further investigations to demonstrate if a correlation exists between BMI and CSA in diabetic patients with CTS. BH was another statistically significant factor between the different groups. It has been proposed that a difference in BH may influence the result of nerve conduction velocity [26], however it is not a known factor to influence the median nerve CSA [27].

In US studies, different CSA cut-off values (9 mm^2^ – 14 mm^2^) at the entry level (inlet) of the carpal tunnel have been reported for CTS confirmation in idiopathic CTS patients [28]. In the present study, we revealed the cut-off values of CSAs measured at different levels of the patients with idiopathic, diabetic and DPN CTS. Although larger CSAs measured in the carpal tunnel 5 cm proximal to the wrist and elbow joint of the median nerve in diabetic and diabetic polyneuropathy patients were reported by Watanabe et al. [11,12], comparative results of measured CSAs at the inlet level of diabetic patients have not been reported before. In the present study, we measured the CSAs within the carpal tunnel in the hands of the diabetic patients with CTS, and the results also showed larger CSAs when compared with the CSAs measured in both the control and idiopathic groups. Several factors are known to be contributing factors to CTS, including mechanical and ischemic factors, external epineurial and perineurial thickening and fibrosis [29], and all of these factors may in-part explain the local enlargement of median nerve CSAs. However, as shown in this study, there may be additional factors contributing to the focal enlargement of median nerve CSAs in diabetic patients, and especially at the level of the inlet. In DM, the polyol pathway, glycation and proinflammatory reactions are known to contribute to the presence of diabetic peripheral nerve injuries [30], and a reduction in myelinated nerve fibers and capillary density may predispose DM patients to the development of CTS [31]. In addition, more ischemic and biochemical changes may further result in the enlargement of median nerve CSAs.

One peculiar finding of the present study is that the measured median nerve CSAs of the patients in the DPN group, especially at *i*1 and *w* levels, were smaller than those measured in the diabetic group. This finding has not been reported previously, but may be partially related to the loss of nerve regeneration capacity in advanced diabetic neuropathy [4,31,32]. This finding may also partially explain why the response to CTS treatment in diabetic patients varies greatly, especially when different intervention methods are used, when compared with the therapeutic results of idiopathic CTS [33-35].

There are limitations to this study. First, some of the patients may have been included twice if they had bilateral CTS symptoms. Second, the number of cases is limited; therefore, US findings in the different subgroups of CTS patients could not be fully analyzed. Further large-scale studies of US findings in CTS among diabetic patients are needed for a better delineation.

## Conclusion

This is the first study to use US and an 8-point measurement method to assess and compare the median nerve CSAs of participants belonging to control, idiopathic, diabetic and DPN CTS groups. We suggest the cut-off values for CTS confirmation in each group, and also show the following: 1) Compared with the controls, the CSAs were significantly enlarged in patients with idiopathic CTS, diabetic CTS and DPN CTS; 2) Compared with the idiopathic group, the CSAs were significantly enlarged in patients with diabetic CTS and DPN CTS; and 3) Compared with the diabetic group, the CSAs were significantly smaller in patients in the DPN group. Several pathophysiologic mechanisms may, at least partially, explain the findings of the inter-group differences of median nerve CSAs. This US 8-point measurement method can be of value as an important complementary tool for the diagnosis of CTS among diabetic patients.

## Misc

Shu-Fang Chen and Chi-Ren Huang contributed equally to this work.

## Competing interests

All of the authors declare no competing or conflicts of interests.

## Authors’ contributions

All authors have read and approved the submitted manuscript. SFC and CRH contributed to the conception and design, data acquisition and analysis, and drafting and revision of the manuscript; CHL, YCC, NWT, and CCC contributed to the conception and design, and clinical data analysis; and WNC contributed to the conception and design, data analysis, and critical revision and final approval of the manuscript.

## Pre-publication history

The pre-publication history for this paper can be accessed here:

http://www.biomedcentral.com/1471-2342/12/15/prepub
